# Three-Dimensional Visualization of Shunt Valves with Photon Counting CT and Comparison to Traditional X-ray in a Simple Phantom Model

**DOI:** 10.3390/tomography10040043

**Published:** 2024-04-12

**Authors:** Anna Klempka, Sven Clausen, Mohamed Ilyes Soltane, Eduardo Ackermann, Christoph Groden

**Affiliations:** 1 Department of Neuroradiology, University Medical Centre Mannheim, Medical Faculty Mannheim, University of Heidelberg, 68167 Mannheim, Germany; 2 Department of Radiation Oncology, University Medical Centre Mannheim, Medical Faculty Mannheim, University of Heidelberg, 68167 Mannheim, Germany

**Keywords:** photon counting, hydrocephalus, shunt, valve

## Abstract

This study introduces an application of innovative medical technology, Photon Counting Computer Tomography (PC CT) with novel detectors, for the assessment of shunt valves. PC CT technology offers enhanced visualization capabilities, especially for small structures, and opens up new possibilities for detailed three-dimensional imaging. Shunt valves are implanted under the skin and redirect excess cerebrospinal fluid, for example, to the abdominal cavity through a catheter. They play a vital role in regulating cerebrospinal fluid drainage in various pathologies, which can lead to hydrocephalus. Accurate imaging of shunt valves is essential to assess the rate of drainage, as their precise adjustment is a requirement for optimal patient care. This study focused on two adjustable shunt valves, the proGAV 2.0^®^ and M. blue^®^ (manufactured by Miethke, Potsdam, Germany). A comprehensive comparative analysis of PC CT and traditional X-ray techniques was conducted to explore this cutting-edge technology and it demonstrated that routine PC CT can efficiently assess shunt valves’ adjustments. This technology shows promise in enhancing the accurate management of shunt valves used in settings where head scans are already frequently required, such as in the treatment of hydrocephalus.

## 1. Introduction

Hydrocephalus, a medical condition characterized by the abnormal accumulation of cerebrospinal fluid within the neurocranium, poses a significant challenge in neurosurgery. To mitigate the potentially severe consequences of elevated intracranial pressure, the installation of a catheter is often required [1]. This surgically placed catheter extends from a ventricle within the brain to the peritoneal cavity, where it facilitates the controlled drainage of cerebrospinal fluid for safe absorption. Central to this drainage system is the shunt valve, a critical component responsible for regulating the flow of cerebrospinal fluid, which is typically located on the side of the patient’s head [2,3].

Conventional imaging modalities are used in the visualization of valves and assessment of their settings. These include a lateral X-ray of the head with the intention of showing the shunt valve in the lateral plane [4,5]. A second option to obtain a readout of a programmable shunt valve is using the manufacturer’s device. In tomographic images, magnetic resonance imaging cannot visualize the shunt valve due to metallic artifacts. However, with computed tomography (CT), parts of the valve can be imaged and analysed.

Patients with a shunt system undergo periodic CT scans to assess the cerebral fluid spaces as a part of the standard surveillance process for hydrocephalus. A previous study categorized the quality of energy-integrated CT devices as not good enough when assessing the settings of the shunt valve proGAV (one of previous models of the proGAV 2.0), even with the implementation of metal artefact reduction algorithms [6]. Our research group has previously explored this intriguing topic, albeit without comparing it to traditional X-ray methods, demonstrating promising results for this in vitro approach [7].

The PC CT overcomes the limitations of CT technology reported during the last decades and allows us to depict even the smallest structures of the middle ear as well as better recognize several materials to create higher-contrast images [8]. This novel approach provides improved image quality and results in clearer images with enhanced tissue differentiation and reduced artifacts [9,10,11]. The cutting-edge technique of photon counting technology, alongside an evolved detector design, addresses the long-standing problems of earlier systems. The impressive system is designed for the purpose of a higher accuracy that could not be achieved in the past. Apart from the fact that it is a new method of improving the accuracy of imaging techniques, it is also very important in the minimization of possible artifacts that might be encountered and thus accurate details of the images will not be obscured. This method leads to an enhanced contrast-to-noise ratio, better spatial definition, and refined spectral imaging capabilities. By employing PC CT, it is possible to diminish radiation dosage, reconstruct images with greater clarity, amend beam-hardening distortions, fine-tune the usage of contrast materials, and forge paths for quantitative imaging in comparison to existing CT technologies. PC CT and Energy-Integrated Detectors (EIDs) exhibit significant differences in their operational mechanisms. Unlike EIDs, which depend on an additional scintillator layer to convert X-rays into visible light, a PC detector directly utilizes a semiconductor layer. When X-ray photons strike the semiconductor, they generate electron–hole pairs that are swiftly separated by the potent electric field, causing electrons to drift towards the anodes. This movement creates an electrical signal captured by an electronic readout circuit attached to the anodes. In this way, a PC detector directly transmutes X-ray photons into electrical signals, with each photon inducing an electrical pulse [12]. Traditional imaging techniques have certain deficiencies due to their own constraints and thus cannot provide necessary information. As a result, this technology became a pathway in the field of medical imaging to counter these limitations. The design of the detectors is a much more sophisticated one, which paves the way for the sharp and highly penetrated images, making the even quicker and more precise diagnosis of diseases possible [13].

This study focused on the three-dimensional possibilities of imaging, as well as the feasibility and effectiveness of PC modality for visualizing programmable shunt valves in order to provide a reliable, efficient, and non-invasive alternative for managing shunt systems in patients undergoing cranial CT for the estimation of cerebral fluid spaces [14,15]. Specifically, two programmable shunt models—the proGAV 2.0 and M.blue (Miethke, Potsdam, Germany) valves—were assessed using a basic in vitro phantom model, so that there was no need to use an additional tool to read the valve assessment form the PC CT scan of the neurocranium. The objective was to eliminate the need for additional tools and simplify valve assessment through PC CT scans of the neurocranium.

Firstly, in this study, imagining in a three-dimensional perspective of two shunt valves—proGAV 2.0 and M. blue—is highlighted. In the second section, a matchup between innovative approaches to imaging techniques and well-known radiological methods with X-ray is comparative analysed.

## 2. Materials and Methods

The study will be summarized on two levels. The first one is built on the three-dimensional visualization of the valve components, and this gives a precise view of its structure. The second level is dedicated to three observers reading and interpreting X-ray and PC CT images along with statistical analysis. This analysis shows the association among inter-rater agreement and the ways in which different imaging methods influence the results. This research intends to give a complete picture of the imaging modalities’ value and the manner in which various observers interpret the images.

The basic phantom setup consisted of an X-ray-translucent spongy material placed on the bottom, and the proGAV 2.0 and M. blue valve (each manufactured by Miethke, Potsdam, Germany) placed on top of it. A measurement tool provided by the manufacturer to assess and verify each setup was utilized. To determine the settings, the charts from the manufacturer’s manual were used as a reference.

### 2.1. Three-Dimensional Visualisation

#### CT Scanners

In the first step of imaging, two energy-integrated scanners were compared, Somatom Definition Flash and Somatom Sensation 64, along with Naeotom Alpha, in a visual test—both scanners manufactured by Siemens Haelthineers (Forchheim, Germany). The imaging parameters of all scanners in this protocol and the signal-to-noise ratio (SNR) are shown on Table 1. These CT scanners were selected due to their availability at the institution and their frequent use in conducting CT examinations.

### 2.2. Comparative Analysis with X-ray to Scrutinize the Feasibility and Efficacy of PC CT in the Assessment of Shunt Valve Adjustments

#### 2.2.1. X-ray

**Figure 1:**
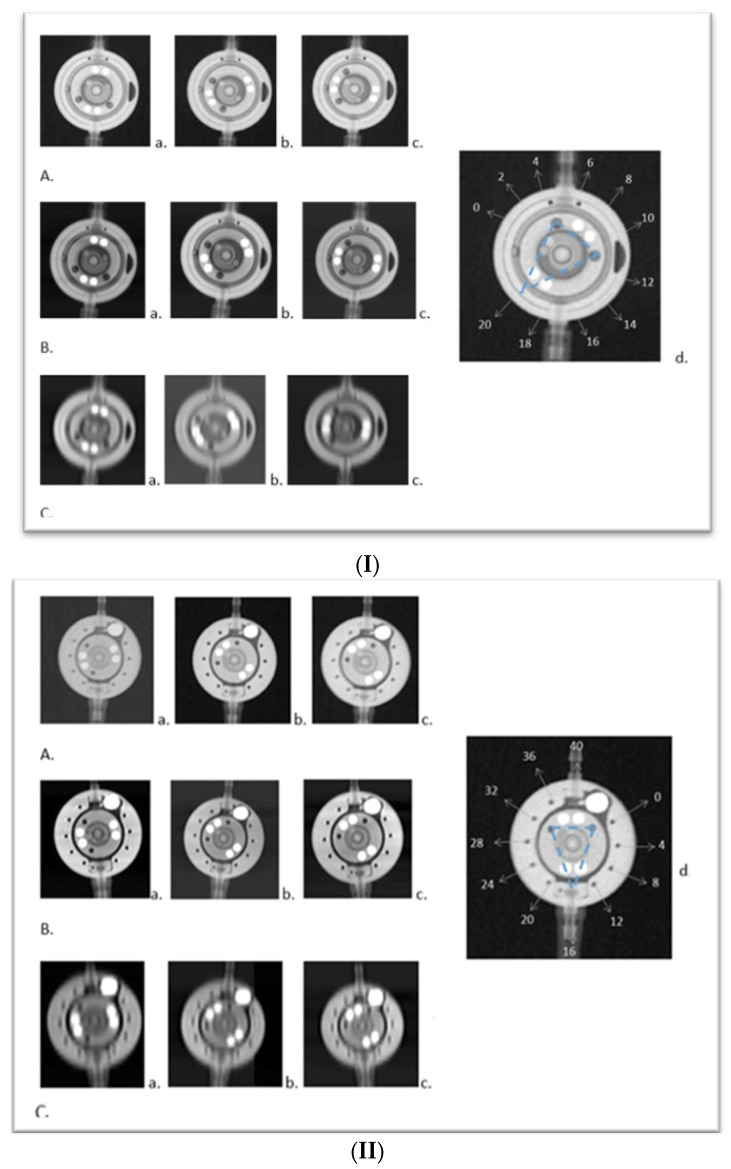
(**I**) **Comparison of X-ray and PC CT of the proGAV 2.0**. Visualization of the proGAV 2.0 valve using X-ray technology (**A**), PC CT with a temporal bone protocol (**B**), and PC CT with a standard cranial protocol (**C**). In each row, from left to right, the valve settings are as follows (readings from a senior observer): (**a**) 6 mmH_2_O, (**b**) 10 mmH_2_O, and (**c**) 12 mmH_2_O. (**d**) Schematic representation of the proGAV 2.0 valve, displaying admittance markings ranging from 0–40 mmH_2_O. Central markings feature a triangular apex, highlighting an adjustment at 20 mmH_2_O (indicated by blue dashed lines). Adapted from “proGAV 2.0: In Touch with You-Instructions for Use”, www.miethke.com, 2 December 2021. (**II**). **Comparison X-ray and PC CT of the M.blue.** Visualization of the M.blue valve using X-ray technique (**A**), PC CT with a temporal bone protocol (**B**), and Photon Counting CT with a standard cranial protocol (**C**). In each row, from left to right, the valve settings are as follows (readings from a senior observer): (**a**) 3 mmH_2_O, (**b**) 10 mmH_2_O, and (**c**) 12 mmH_2_O. (**d**) M.blue: Schematic representation of the shunt imaging featuring a coding burrhole gravitational unit used for reading the setting. Centralized triangular markings indicate the encoded adjustment level, set here to 16 mmH_2_O (illustrated by blue dashed lines). Adapted from “M.blue: The Balanced Way of Life-Instructions for Use”, www.miethke.com, 24 March 2022.

The traditional X-ray imaging was conducted with 70 KV, 16 mAs, using the same basic phantom; the pictures were presented with a window level (WL) mean of 2270 +/− 618 and window width (WW) of 2168 +/− 209 (Figure 1I(A),II(A)).

#### 2.2.2. Photon Counting Computer Tomography

To acquire PC CT images, we used a Naeotom Alpha (Siemens Healthineers, Forchheim, Germany). The phantom was carefully positioned on the CT table, occupying the designated area for the patient’s head. Subsequently, two consecutive scanning spiral protocols were applied: a cranial CT protocol (120 KV, quality reference 72 mAs, ME67, pitch factor 0.35, rotation time 0.5 s, and matrix size 512 × 512) (Figure 1I(C),II(C)) and a temporal bone CT (with the difference in quality reference 25 mAs and pitch 0.85 in comparison to the cranial protocol—Figure 1I(B), II(B).

Special focus was given to preparing the best reconstruction setting possible by adjusting the windowing and layer thickness to mimic the quality of the X-ray imaging with PC CT imaging. The reconstructions were performed using the Syngo.via Client 8.3 software (Siemens Healthineers, Forchheim, Germany). For the reconstruction from temporal bone protocol, the settings were as follows: Hr84: Q2, 4 mm, increment 0,1 mm; proGAV2.0 WL 34,859 +/− 5356 WW 109,17/2194; M.blue WL 21,784 +/− 11,094, WW 6663 +/− 4108. For the cranial protocol reconstructions, the following settings were applied: Hr68, 4 mm, increment 0.1 mm; proGAV2.0 WL 46,884 +/− 17,665 WW 16,296 +/− 5542; M.blue WL 20,526 +/− 4540, WW 7022 +/− 2182.

#### 2.2.3. Observer Readings and Levels of Confidence

Three independent observers—two radiologists with approx. 10 years of clinical experience each and one junior radiologist in training—described 30 valve settings (mmH_2_O): 15 of each valve as well as their level of confidence using the following grading system (range 1 to 4, from the lowest to highest confidence level); i.e., (1) I am uncertain or unable to recognize neither the valve nor its setting, (2) I am confident enough to identify the type of valve, but not the setting, (3) I am confident enough to include my measurement in the written report, (4) I am absolutely confident in the measurement. The evaluation was conducted in a random order, and the type of the reconstructions was blinded to observers.

#### 2.2.4. Statistical Analysis

For all imaged valve settings, Fleiss’ Kappa for inter-rater agreement was evaluated using pyirr library. The levels of confidence were also presented, and a one-way analysis of variance (ANOVA) was conducted on the results of each observer to determine if the differences between the methods were also statistically significant.

## 3. Results

### 3.1. Three-Dimensional Visualisation

**Figure 2:**
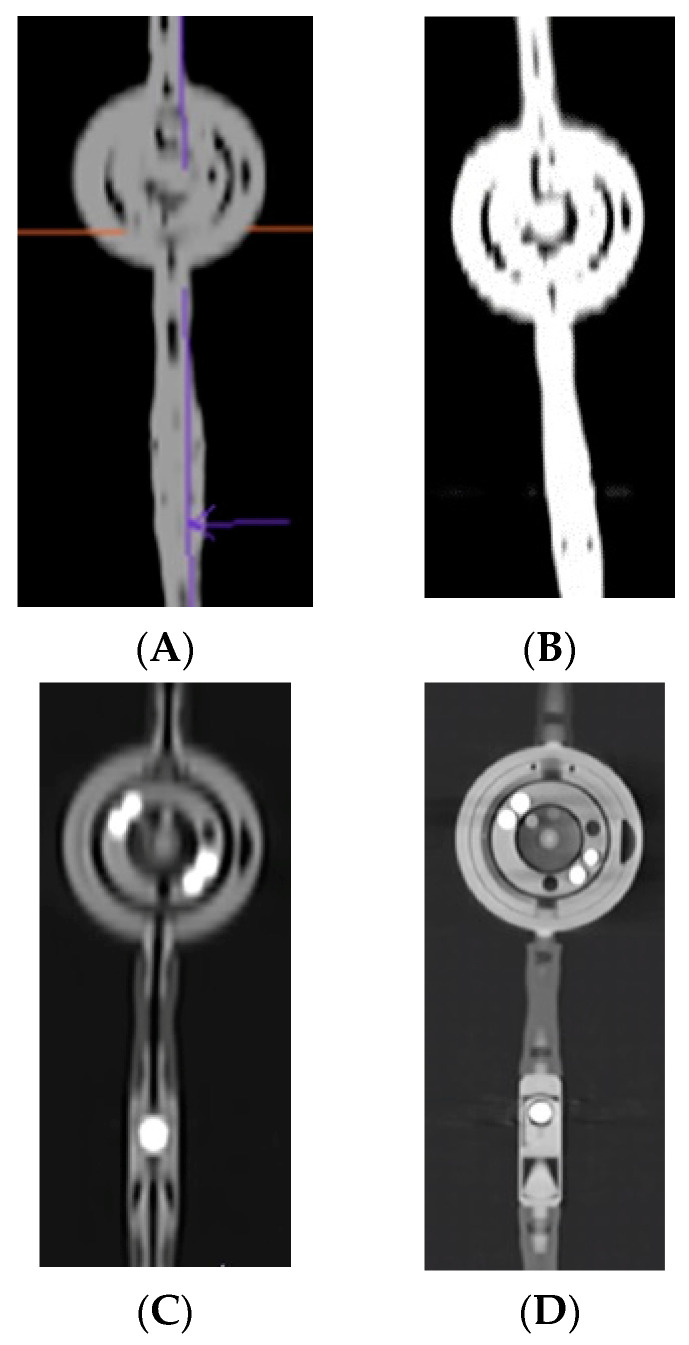
**Comparison of imaging for the proGAV 2.0.** An example of imaging the proGAV 2.0 to illustrate the differences between energy integrated detectors, namely, (**A**) Somatom Definition Flash, (**B**) Sensation 64 Siemens and Photon Counting Naeotom Alpha (**C**) with the same clinically used cranial protocol, sharp kernel reconstruction with the thinnest layer of scan, prepared by an experienced radiologist. Additional imaging with detailed protocol in Photon Counting CT with sharp edges by using a higher pitch (0.85) as in clinically applied temporal bone protocol (**D**). The comparison of imaging techniques for the proGAV 2.0 valve provides valuable insights into the capabilities of different detectors. The clarity and detail achieved through PC CT, as demonstrated (**C**,**D**). The sharp edges and high resolution offer a level of precision essential for accurate diagnosis and treatment planning.

The initial phase of the visualization process revealed that there was no better CT imaging technology available at our institution for the 3D imaging of small objects like a shunt (approximately of a coin size). A comparison was conducted involving two scanners manufactured by Siemens Haelthineers (Forchheim, Germany)—energy-integrated (Somatom Definition Flash and Somatom Sensation 64 Figure 2A,B) and PC CT (Naeotom Alpha Figure 2C)—under clinically comparable conditions with cranial CT protocols. The visual results clearly favoured the Naeotom Alpha Siemens scanner due to its significantly superior imaging quality of the shunt valve (Figure 2D). The utilization of PC CT technology also provided a broader spectrum of radiological grayscale in the images, further enhancing the diagnostic capabilities, as well as better visualisation and signal to nose ratio transferring into better resolution with lower dose acquisition.

**Figure 3:**
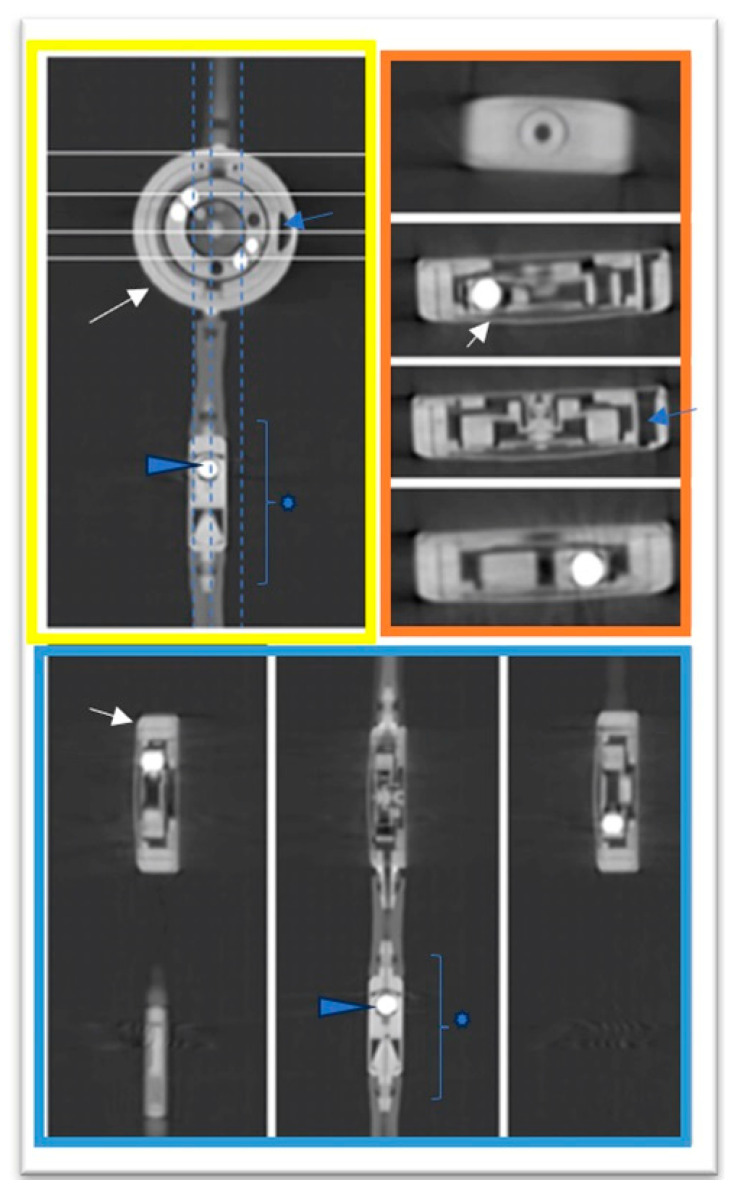
Three-dimensional imaging of proGAV 2.0. Yellow frame: coronal view of proGAV 2.0 valve with axial lines (white) and sagittal lines (dashed vertical) for reference; orange frame: axial layers of the shunt valve at 0.5 mm intervals, illustrating parallel layers from top to bottom; blue frame: sagittal layers at 0.5 mm intervals, revealing lateral layers from left to right in the coronal image of proGAV 2.0. Tantalum weight (arrowhead), valve marking (blue arrow), adjustable differential pressure unit (white arrow), and gravitational unit (star). Description of the valve parts in accordance with the manufacturer’s Miethke description: “proGAV 2.0: In Touch with You-Instructions for Use”, www.miethke.com, 2 December 2021.

**Figure 4:**
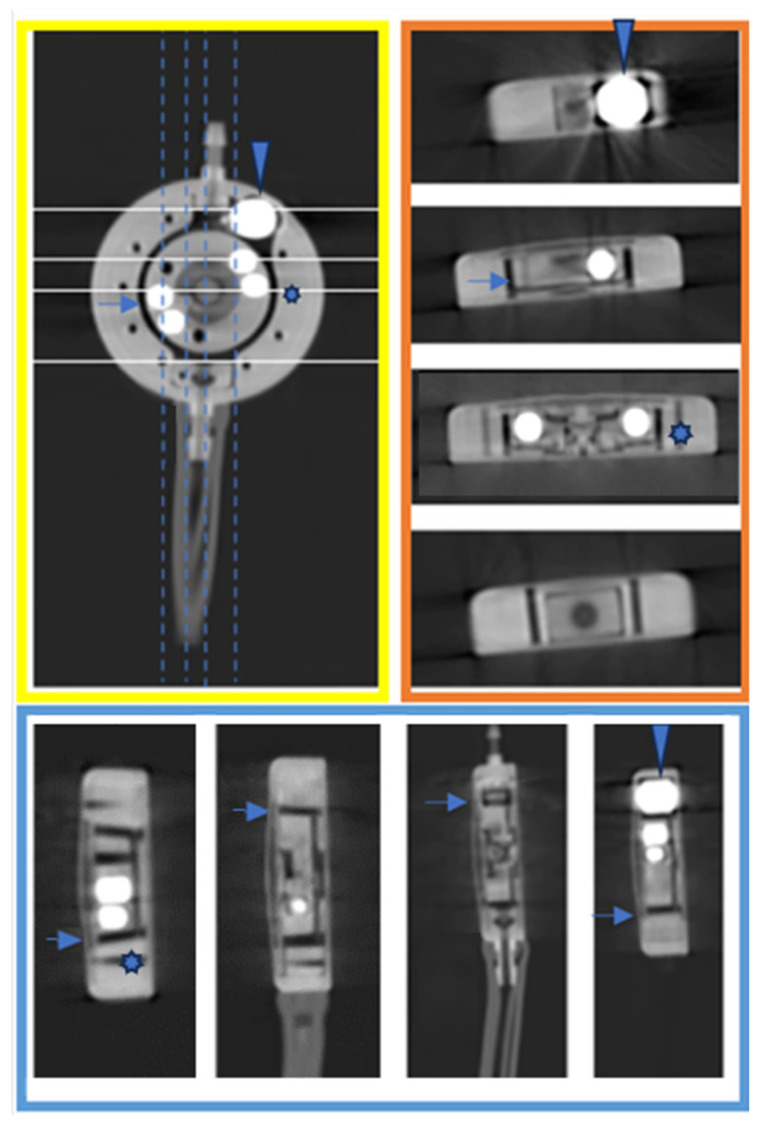
Three-dimensional imaging of M.blue valve. Yellow frame: coronal view of M.blue valve with axial lines (white) and sagittal lines (dashed vertical) for reference; orange frame: displays axial layers of the shunt valve at 0.5 mm intervals, illustrating parallel layers from top to bottom; blue frame: sagittal layers at 0.5 mm intervals, revealing lateral layers from left to right in the coronal image of M.blue. Tantalum weight (arrowhead), rotor rim (blue arrow), and one of the coding burrholes of the gravitational unit (star). Description of the valve parts in accordance with the manufacturer’s Miethke description: “M.blue: The Balanced Way of Life-Instructions for Use”, www.miethke.com, 24 March 2022.

It was decided not to solely compare individual scanners due to lack of possibilities to read the valve, as already confirmed in the literature, and mentioned in the introduction. To demonstrate the superior artifact-free 3D results of the proGAV 2.0 and M.blue shunt valves (Figure 2D, Figure 3 and Figure 4) the imaging was supplemented by a clinical protocol typically employed for middle-ear imaging, with a higher pitch in comparison to cranial CT. In Figure 3 and Figure 4, three-dimensional imaging is showcased, offering an unprecedented level of detail and clarity regarding the shunt components, as outlined in the respective figure legends.

### 3.2. Comparative Analysis with X-ray to Scrutinize the Feasibility and Efficacy of PC CT in the Assessment of Shunt Valve Adjustments

In the second level of this study, we demonstrated that valve adjustments can be accurately assessed using the cranial protocol. Additionally, the phantom model yielded even superior visualization of the shunt in the middle-ear protocol, showcasing excellent results, as illustrated on examples from Figure 1I,II.

There was a significant inter-rater agreement among the three assessors for both shunt valves assessments, with a Fleiss’ Kappa value of 0.551, signifying a substantial level of agreement across all 30 settings. The study revealed that when using the temporal bone protocol, all observers were absolutely confident by describing the valve of both shunts. The ANOVA analysis showed a total variation of 83,953, with 2 degrees of freedom, an F-statistic 1.171, *p*-value of 0.315. The results suggest that there is no statistically significant difference in outcomes across the various methods at the 95% confidence level. The *p*-values associated with the method factor consistently exceeded the 0.05 threshold. This result suggests that, along with X-rays, CT scans in cranial protocols should be considered appropriate for assessing adjustments, potentially eliminating the need for additional X-rays.

## 4. Discussion

The presented study evaluates opportunities for the application of PC CT execution and the solving some of the old imaging problems [15,16]. The shunt valve imaging with CT has been investigated in a study carried out by Slonimsky et al. [6]. They utilized energy integrated detectors, their readings were limited and the proGAV setting between 0 and 10% was not acceptable. Another study directly examining the feasibility of CT imaging to display shunt valves was by Decramer et al. [17]. The predictions of accuracy the settings varied, according to them, and failed to specifically look at the proGAV, but it did show positive results with the Codman and Sophysa valves using the neuronavigation software.

Our study addresses a novel imaging of PC CT in context of hydrocephalus treatment, as we partially showed in one of our posters in 2023 [7]. One of the limitations of our study was the simple phantom setting; however, it fulfilled its role because the main goal of this work was to show new possibilities of the scanner. The second limitations of our study is that we examined a relatively small number of valves, exclusively from one manufacturer. However, it is noteworthy that these valves were never effectively visualized using CT scanners.

It is also worth pointing out that the positive outcomes of the test suggest that the cranial protocol could be of great use in cases when a fast evaluation of shunt assessments is required. The valve can be sufficiently imaged and due to aspects concerning radiation does; even if the images of cranial protocol might have been slightly less sharp, all viewers confirmed that they were still clear enough to check for proper valve assessment. Also, the readings are recorded and included in the CT scan and can be also included in physicians’ report for the patient and speed up the diagnostic process. Additional lateral X-ray is rendered redundant and thus this approach could help reduce radiation exposure [18].

This is a preliminary study highlighting an immediate medical potential in diagnostics and as such further investigation is required to fully explore the technical spectrum of possibilities that this technique may offer. The further benefits of PC CT technology, such as an increased contrast-to-noise ratio and an improvement in spatial resolution, have been researched to some extend [19]. There are numerous possibilities yet to research in order to gain intricate insights into the morphology and functionality of scanned objects [20]. Other shunt valves can possibly be imaged in great detail, and further studies may reveal key insights from other manufacturers. Future studies exploring these possibilities across different manufacturers could uncover valuable insights into device performance, durability, and potential complications, thereby advancing patient care and medical device innovation.

Our study opens up possibilities for visualizing the internal components of the valve, which could potentially aid in identifying recognizable diagnostic malfunctions, as suggested by Bettag et al., 2022 [21]. It is noteworthy that a significant clinical concern pertains to the duration until revision and the longevity of the implant [22]. Our study suggests that the accuracy demonstrated in our research could be valuable in identifying potential causes of revisions, such as flow blockages in the valve or for in vitro imaging for forensic purposes.

It is important to highlight that, unfortunately, the PC scanner is not a widely adopted technology at present, and there are relatively few scanners of this type around the world. This scarcity could be attributed to factors such as high costs, technological complexity, and a lack of widespread expertise in operating such devices. However, considering the significant advantages that photon counting offers, such as higher image resolution, better contrast, and the potential for lower doses of radiation compared to conventional imaging methods, it is reasonable to anticipate a gradual increase in the adoption of this technology. The benefits could drive increased research, development, and eventually, broader implementation of photon counting scanners As the technology matures and becomes more accessible, and as the medical community becomes more familiar with its operation and advantages, it is plausible that PC could become a new standard in medical imaging and possibly other areas requiring high-precision imaging. Additionally, the integration of PC technology into existing medical imaging modalities could streamline workflows, reduce scan times, and enhance the overall efficiency of imaging. Our research also touches on the topic of artifacts during the imaging of disease entities and related artifacts. Artifacts can be reduced by appropriate reconstruction, but they still pose a challenge in imaging. The way in which a CT scan is conducted has an impact on the image quality, which will be subject to reconstruction. Other authors also note significant advantages associated with the use of photon counting CT in this context [23]. PC CT is known for its superior contrast resolution and energy discrimination capabilities. While artifacts remain a challenge in medical imaging, the introduction and development of photon counting CT technology represent a promising advancement in overcoming these issues. The unique properties of photon counting detectors provide a pathway to significantly improved image quality, reducing artifacts and potentially transforming CT imaging into an even more powerful diagnostic tool [24,25,26].

Hence, our study shows that the PC CT may be able to provide a precise visualization of two selected programmable shunt valves for the treatment of hydrocephalus. The high performance of PC CT when compared to the conventional X-ray method suggests that it could have a significant role in clinical practice. Nevertheless, the importance of conducting additional studies, including clinical trials, cannot be overstated. Such research is critical to validate our findings further and could serve as a foundation for the broader application of this technology in healthcare settings. The potential of PC CT to revolutionize the visualization of medical implants and improve treatment outcomes for patients with hydrocephalus is immense, but its widespread adoption will depend on a deeper understanding and confirmation of its benefits through rigorous scientific scrutiny.

## 5. Conclusions

In conclusion, our study highlights the promise of PC CT in visualizing of two selected programmable shunt valves for hydrocephalus treatment. The performance of PC CT compared to traditional X-ray methods suggests that it could become a valuable asset in clinical practice. However, further research, including clinical trials, is necessary to validate these findings and pave the way for its widespread adoption in healthcare.

## Figures and Tables

**Table 1:** Comparison of the technical aspects of scanners used for clinical purposes as well as for our simple phantom setup. In all cases, the cranial CT scan parameters clinically used for each scanner were employed. Notes: SNR—the signal-to-noise ratio—was calculated by measuring Hounsfield Units (HU) in a 15 mm^2^ area of the scan always positioned in front of the valve chamber and dividing it by the standard deviation. Please note the exposure per slice was also the lowest in the Photon Counting CT CTDI (computed tomography dose index).

	EID ^1^	EID ^1^	PC CT ^2^
Computed Tomography Scanner	Sensation 64	Somatom Definition Flash	Neaotom Alfa
KV	120	120	120
mAs (quality ref.)	355	292	71/25 *
Pitch Factor	1.01	0.55	0.35/0.85 *
Collimation	14.2	12	57.6
Exposure time per rotation	1	1	0.5
CTDI	54.99	54.6	39.5
Acquisition	sequential	spiral	spiral
SNR	4	5.9	7

^1^ Energy-integrated detectors. ^2^ Phonon counting. * Temporal bone protocol.

## Data Availability

The data presented in this study are available on reasonable request from the corresponding author.
